# Medical News and Miscellany

**Published:** 1899-02

**Authors:** 


### Medical News and Miscellany.

**Whom Miles** would destroy he first makes mad.

**Dr. W. L. Lacey** has removed from San Antonio to Austin.

**Dr. H. Wolff** has removed from Marion, Tex., to Seguin, Tex.

**Dr. S. M. Jenkins** has removed from Summers' Mills to Waco.

**Dr. A. J. Gray** has removed from Grand Saline to Wills Point.

**Dr. W. L. Lacy** has removed from San Antonio to Austin, Texas.

**Dr. Frank Paschall** has been appointed city physician of San Antonio.

---

**Dr. D. M. Cook,** Louisville Medical College, 1898, has located at Jonah, Tex.

---

**Dr. R. M. Harkey** has removed from Caddo, Mills, Tex., to Little Rock, Ark.

---

**Dr. W. F. Blunt,** of Lockhart, has been re-appointed State Health Officer of Texas.

---

**Surgeon H. R. Carter**, M. H. S., has been made inspector of all Cuban ports except Santiago.

---

**Dr. Sam C. Ball** has removed from New Boston to Dallas, and has opened an office at Elm and Central Avenues.

---

**Anatomical Bill.**—The Texas Legislature now in session has passed an act legalizing the dissection of bodies unclaimed.

---

**Wanted.**—Physician to buy residence and take charge of practice at once. Address: A. A. Jones, M. D., Holliday, Texas.

---

**Dr. L. D. Hill,** of Webberville, Texas, an old Confederate soldier, has been appointed surgeon to the Texas Confederate Home at Austin.

---

**Dr. W. E. Davis** (colored), of Fort Worth, has been convicted of criminal abortion and given two years in the pen. New trial asked for.

---

**Dr. Frank Ross,** of Bryan, Tex., son of the late ex-Governor Ross, has been appointed assistant physician to the State Insane Asylum at Austin.

---

**Dr. R. B. Anderson** has removed from Seguin to Sherman, Texas. He has just returned from New York, where he took a course at the Polyclinic.

---

**Dr. E. E. Guinn,** of Jacksonville, has been appointed physician to the penitentiary at Rusk, and Dr. W. E. Fowler, of Bastrop, to that at Huntsville.

**Epileptic Asylum.**—The Texas Legislature has passed a bill providing for an epileptic asylum at Abilene, Texas. This was a "platform demand."

---

**The delay** in this issue was caused by a rush of State work which overtaxed the resources of our printers for awhile. We ask the indulgence of our patrons.—Eds.

---

**Dr. Vard H. Hulen,** late of Galveston, has located in San Francisco instead of New York, as announced in our December number. His address is 406 Sutter street.

---

**Hamilton's Successor.**—Dr. Geo. H. Simmons, of Lin coln, Neb., has been elected editor of *Journal of the American Medical Association* vice the lamented Hamilton.

---

**Dr. J. L. Dillard,** a leading physician of Richmond, Texas, died February 10th (inst.) from septicæmia, the result of an accidental pricking of his hand during a surgical operation.

---

**Dr. I. J. Jones,** the retiring surgeon of the Confederate Home, has resumed practice in Austin. Dr. Jones is credited with very efficient and satisfactory service during his incumbency of four years.

---

**Dr. Fred Hadra,** of San Antonio, who was surgeon of the First Texas Regiment (which didn't get a chance to remember the Maine), is now Assistant Surgeon First U. S. Regulars, and is in Havana.

---

**Compulsory Vaccination in Cuba.**—Chief Surgeon Maus, of the Seventh Army Corps, has asked General Fitzhugh Lee, its commander, to make the vaccination of every one in the province of Havana compulsory. One hundred and sixty-one cases of smallpox have been reported.

---

**Dr. F. C. Ford,** of Nacogdoches, Texas, long time medical director of Camp Mabry, T. V. G., has been commissioned Surgeon in the United States Army, after examination by the Army Board. We learn that Major Ford is on the staff of General Lee at Havana. There is not an abler surgeon nor a better man in the service, and General Lee isthe one to be congratulated.

**Wanted.**— Agents for "History of the Spanish-American War," by Henry Watterson. A complete, authentic history; illustrated with over 76 full-page half-tones and many richly colored pictures. Large royal octavo volume, superb outfit, postpaid for only 50 cents (stamps taken). Most liberal terms given. The greatest opportunity of the year. Address: The Werner Company, Akron, Ohio.

---

**A High Flyer.**—In keeping with the handsome new fast train service to the North and East inaugurated by the I. & G. N. R. R. in December and now in very successful operation, a beautiful chromo-lithograph calendar, for the year 1899, entitled "A High Flyer," is issued and will be mailed free on application. D. J. Price, G. P. A., Palestine, Texas, or P. J. Lawless, P. A., Austin, Texas. It is a work of Art.

---

**The Journal of the American Medical Association.**— At a meeting of the Board of Trustees of the American Medical Association, held in Chicago, January 2d, the selection of an editor to succeed the late Dr. John B. Hamilton was postponed until a future meeting, and in the mean time the *Journal* will be issued under the direction of Dr. Truman W. Miller, Chairman of the Publication Committee of the Association.

---

**Dead Again!**—Judiciary Committee No. 2 (House), reported adversely on Shropshire's bill to create a State Board of Medical Examiners. That is the bill, a copy of which was sent us by physicians in North Texas, and called the "Illinois Bill," which was published in our December number. The State Association's Bill was introduced in the Senate on the 21st by Senator Yett and referred to the proper committee. No report at this writing.

---

**Many suicides** have recently been recorded in and about the metropolis. It is a matter of some wonder that so many seemingly intelligent persons elect to depart with the aid of carbolic acid, a most distressing way it would seem, although eminently antiseptic. The man who took a large dose of laudanum and then pulled down the lid of a trunk, hermetically sealing himself up in his own carbonic-acid gas, surely had a more comfortable time of it.

**The Tax on Doctors.**—February 24, the occupation tax question came up in the Legislature. The grain dealers and others want to be exempt, as well as the doctors. It is in this way that all efforts by the medical profession are paralyzed. The State Association of Barbers want a bill to regulate the practice of "barbery." The subject of a revision of the occupation tax law will be left to a commission. So, we suppose, that's the last of the doctors' prayer for relief from "occupation" tax.

---

**Quarantine Appointments.**—Dr. T. J. McFarland; Quarantine Officer at Port Lavaca; Dr. A. S. Wolff, Quarantine Officer at Brownsville; Dr. W. E. Pugh, Quarantine Officer at Rockport; Dr. W. M. Yandell, Quarantine Inspector at El Paso; Dr. Malone Duggán, Quarantine Inspector at Eagle Pass, vice Dr. A. H. Evans; Dr. J. M. McKnight, Quarantine Inspector at Laredo, vice Dr. T. J. Turpin. The four first named are the old officers of previous administrations. At this writing no appointments have been made for Galveston, Sabine Pass and Velasco.

---

**Dr. Harrison,** of Columbus, who is chairman of the State Medical Association's Committee on State Board of Health, with two members of his committee, had an interview with the Governor soon after his inauguration, and discussed sanitation. The Governor expressed himself as "impressed with the importance of the subject," and asked the committee to go home and formulate their ideas of a bill and submit it, and he would "think about it." The committee have prepared a bill and will again call on his excellency this week (last week of February), then the bill will will be introduced in the Senate by Senator (Dr.) Yett of Burnet. After which "comes the judgment." "Hope springs eternal" in the medical breast.

---

**Supervising Surgeon-General Wyman,** of the marine hospital service, is sending out a circular letter to the counties and corporate towns of the States asking for a report of the vital statistics thereof for the year 1898. Unfortunately the distinguished statesmen who framed the laws of Texas have never been able to appreciate the necessity for the preservation of any such record as this, and consequently our statutes are not encumbered with any provision for that purpose. It is s noteworthy fact that the surgeon-general, in this letter, desires specific information upon all those forms of disease indigenous to every State which are generally regarded as preventable. Possibly if the surgeon-general would send a copy of this letter to each member of the Legislature, it might arouse their perceptions to an approximate conception of its importance.—*Colorado Citizen.*

---

**Hypodermic Medication.**—

| | |
|---|---|
| Creosote | 25 |
| Ol. oliv. sterilis., | |
| or Ol. amygd. dulc.; | |
| or Vaselin liq. | 100 |
| Or: | |
| Guaiacol | 10 |

Subcutaneously in pulmonary tuberculosis.—Eloy.

*Caution.*—Not to be used in congestion, hæmoptysis, renal lesions and pyrexia.

| | |
|---|---|
| Camphor | 2 |
| Liq. paraffin | 8 |

M.: S. A syringe (one c.c.) contains twenty centigrams camphor.

---

**Hypodermic Solutions.**—

| | |
|---|---|
| Creosote, pure | 1 gm. |
| Aseptic olive oil | 14 gm. |
| Iodide of iodoform | 1 gm. |
| Olive oil | 29 gm. |
| Crystals of phenic acid | 1 gm. |
| Olive oil or water with a little alcohol | 49 gm. |
| Neutral hydrochlorate of quinine | 1 gm. |
| Distilled and boiled water | 9 gm. |
| Antipyrin | 1 gm. |
| Distilled water | 10 gm. |
| Ichthyol | 10-20 gm. |
| Acqua destil | 100 gm. |
| Sod. arsenit | gr. ii. |
| Aquæ destil | ʒi. |

Dose: Two or three drops.

---

**The Association's Bill to Regulate Practice.**—This bill provides for three separate boards of examiners; one for physicians, one for homo's and one for eclectics—each board to be represented on a medical council consisting of the President of the University Regents and some State Department officers and the three representatives aforesaid. The committee having the bill in charge, consisting of Dr. J. T. Wilson—author of the bill—President of the State Association, Dr. M. D. Knox of Hillsboro, Dr. Scott of Temple, Dr. A. B. Gardner, of Bellville, Drs. A. N. Denton and M. M. Smith, of Austin, had an interview with the House committee February 18th and discussed the bill. Dr. A. C. Oliver, representative from Cass county is chairman of the the House committee in whose hands the bill now is. There are two other doctors on this committee. The homo's were represented at the interview by a "Dr. Gordon;" a flashy person with a plug hat and diamonds—said to be some sort of a "healer" represented—well—himself and all the unrepresented element of irregularity I suppose. We understand that these last mentioned persons "'lowed" that if the "medical council" part of the bill were omitted they would have "nothin agin the bill." A member of the Association's committee stated after the interview that he thought the House committee were favorably impressed and that they would make a favorable report.

---

**Disinfection for Smallpox.**—Apartments infected by smallpox shall be disinfected by one or both of the following methods:

(*a*) Exposure to sulphur dioxide for twenty-four to forty-eight hours.

(*b*) Washing with a solution of bichloride of mercury 1-100, or a 5 per cent. solution of pure carbolic acid.

Clothing, bedding and articles of furniture exposed to the infection of small-pox shall be disinfected by one or more of the following methods:

(*a*) Exposure to sulphur dioxide for twenty-four to forty-eight hours.

(*b*) Immersion in a solution of bi-chloride of mercury 1-1000, 5 per cent. solution of pure carbolic acid.

(*c*) Exposure to steam at a temperature of 100 to 102° C. for thirty minutes after such temperature is reached.

(*d*) Boiling for fifteen minutes, the articles to be completely submerged.

[NOTE.—Formaldehyd gas may be used when applicable.—*M. H. S. Reports.*

---

**At the regular annual meeting** of the Tri-State Medical Association of Mississippi, Arkansas and Tennessee, held in Memphis, December 20th, 21st, and 22nd, 1898, the following resolutions were adopted:

WHEREAS, the medical laws of the various States have been so perverted by political influence as to give legislative sanction to grotesque, ignorant and dangerous sects of pretenders and charlatans; and

WHEREAS, the privileges granted to one of the most outrageous aberrations, namely, the so-called Osteopathy, constitute a disgrace to the States in which the "Osteopathies" are legally intrenched; and

WHEREAS, a certain William Smith, Osteopathist, having been roundly denounced, together with his sect, by Parke, Davis & Co., and the *Medical Age*, now brings suit against both for $25,000 damages; therefore

Be it declared the sentiment of the Tri-State Medical Associatiov of Mississippi, Arkansas and Tennessee, that Parke, Davis & Co. and the *Medical Age* are entitled to the sympathy of its members and of all medical practitioners; that we wish and expect them to enjoy a complete triumph in repelling this legal assault; and that wheresoever a powerful house takes a bold stand in opposition to quackery it promotes the interests of legitimate and honorable medicine and the welfare of humanity.

### Smallpox and Sanitation.

The statement so persistently made by some of the opponents of vaccination, that the decline of small pox during this century is due to improved sanitation has attracted much attention and has been much relied on by those outside medical circles to prove that vaccination has had nothing to do with the improved condition of affairs. It is important then to observe, as is ably shown by Walter Lloyd in the *Westminster Review,* that if, instead of comparing the present state of affairs with that which held good at the beginning of the century, we enter into a more detailed examination of the different periods, we find that during the middle period the sanitary condition of London underwent great deterioration in consequence of the rapid growth of the population, and that during this period both the general and zymotic mortality were considerably increased; in fact the general death rate became "at least twenty-five per cent. higher than it was between 1820 and 1830." Yet during this insanitary period the smallpox continued to decline, showing how little insanitation had to do with the prevalence of the disease.—*The Hospital.*

